# Retraction Note: Gene-gene interaction network analysis of ovarian cancer using TCGA data

**DOI:** 10.1186/s13048-015-0146-2

**Published:** 2015-03-26

**Authors:** Huanchun Ying, Jing Lv, Tianshu Ying, Shanshan Jin, Jingru Shao, Lili Wang, Hongying Xu, Bin Yuan, Qing Yang

**Affiliations:** Department of Gynecology and Obstetrics, Shengjing Hospital of China Medical University, No.36, Sanhao Street, Heping District, Shenyang, Liaoning Province 110004 China; Department of Oncology, The fifth Hospital of Shenyang, Shenyang, 110023 China; Department of Gynecology and Obstetrics, The ninth Hospital of Shenyang, Shenyang, 110024 China

## Retraction

The Publisher and Editor regretfully retract this article [1] because the peer-review process was inappropriately influenced and compromised. As a result, the scientific integrity of the article cannot be guaranteed. A systematic and detailed investigation suggests that a third party was involved in supplying fabricated details of potential peer reviewers for a large number of manuscripts submitted to different journals. In accordance with recommendations from COPE we have retracted all affected published articles, including this one. It was not possible to determine beyond doubt that the authors of this particular article were aware of any third party attempts to manipulate peer review of their manuscript.
